# Dynamic Source Routing Strategy for Two-Level Flows on Scale-Free Networks

**DOI:** 10.1371/journal.pone.0082162

**Published:** 2013-12-12

**Authors:** Zhong-Yuan Jiang, Man-Gui Liang, Jia-Jing Wu

**Affiliations:** 1 Institute of Information Science, Beijing Jiaotong University, Beijing, People’s Republic of China; 2 Beijing Key Laboratory of Advanced Information Science and Network Technology, Beijing, People’s Republic of China; 3 Department of Electronic and Information Engineering, The Hong Kong Polytechnic University, Kowloon, Hong Kong; King Abdullah University of Science and Technology, Saudi Arabia

## Abstract

Packets transmitting in real communication networks such as the Internet can be classified as time-sensitive or time-insensitive. To better support the real-time and time-insensitive applications, we propose a two-level flow traffic model in which packets are labeled as level-1 or level-2, and those with level-1 have higher priority to be transmitted. In order to enhance the traffic capacity of the two-level flow traffic model, we expand the global dynamic routing strategy and propose a new dynamic source routing which supports no routing-flaps, high traffic capacity, and diverse traffic flows. As shown in this paper, the proposed dynamic source routing can significantly enhance the traffic capacity and quality of time-sensitive applications compared with the global shortest path routing strategy.

## Introduction

In the past two decades, a variety of real-world networks are demonstrated to have small-world phenomenon [1] and scale-free properties [2], [3], such as the Internet, World Wide Web (WWW), highway networks, social networks, etc. The dynamic traffic processes of network have attracted much research interest. In order to improve the traffic capacity of the complex communication networks, a lot of research has been done in three aspect: designing efficient routing strategy [4]–[25], optimizing network strucure [26]–[31] and reallocating the finite network resources [32]–[40] such as node’s delivering capacity, link’s bandwidth, and queue resources. Compared with the high implementation cost of changing network structure and reallocating finite network resources, developing a better routing strategy is preferable to improve the network traffic capacity.

In the global dynamic routing strategy [22], the path between any source node 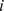 and destination node 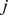 is defined as the path with minimum sum of queue length of nodes, which is denoted as
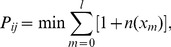
(1)where 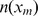 is the queue length of node 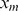 and 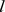 is the path length. Because global dynamic routing strategy considers the real-time queue length of nodes as routing costs to dramatically redistribute the overall traffic on all nodes, the traffic capacity of network achieves the highest value among recently proposed routing strategies so far [22].

However, the global dynamic routing enhances traffic capacity of network at the cost of longer packet traveling time from source to destination, and might cause routing flaps in real communication networks. Further more, several kinds of applications in the Internet such as video conferences, telephone communication are quite time-sensitive, while some other such as email services are relatively time-insensitive which can tolerate longer packet traveling time. Therefore, in this paper, we introduce two different kinds of traffic flow in the traffic model which is not considered in previous studies. To guarantee the efficiency of time-sensitive applications, we divide all flows in the network into two levels: the first is time-sensitive and the other one is time-insensitive. In order to optimize the packet transportation process in this two-level flow traffic model, we propose one dynamic source routing(DS), in which each data packet carries the complete path information from its source to destination. The explicit path of each packet is computed once the packet is generated. Intermediate nodes need not to keep routing table, and no routing flaps occur any more under this expanded global dynamic routing strategy.

This paper is organized as follows. In the following section, the network model, two-level flow traffic model, and the dynamic source routing strategy for two-level flows are described in detail. Then the simulation results are presented and discussed. The paper is concluded in the last section.

### The Models and the Routing Strategy

Empirical studies indicate that many real complex network structures such as the Internet and the WWW are heterogeneous with a power-law degree distribution 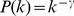, and many networks models have been raised. Among them, the Barabási-Albert (BA) network model with 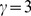 is well-known and widely used. Without loss of generality, we adopt the BA network model in this paper. The construction of a BA network is as follows. Starting from 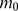 fully connected nodes, at each step, a new node with 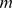
(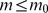) edges are added to the existing network, and the other end of each new edge is chosen preferentially according to the probability
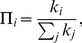
(2)where 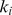 is the degree of node 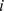, and 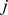 runs over all existing nodes in the network. The growth process of BA scale-free network is terminated once the number of total nodes in the network reaches the scale we need.

Network model is the infrastructure on which the traffic dynamics take place. Traffic model reveals the dynamic running progress of traffic on the network model. To better support distinguishing traffic flows on BA model like networks, here we present one two-level flow traffic model which is described as follows. Each node is considered as both host and router to generate packets or forward packets. Each node can deliver at most 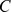
(here, we set 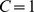) packets at each time step to its immediate neighbors and maintains two queues, one for level-1 packets and the other one for level-2 packets. At each time step, 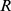 packets are generated in the network with randomly chosen sources and destinations. A fraction of these 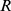 packets are labeled with level-1 as the time-sensitive packets, the others are time-insensitive ones with level-2. Level-1 packets are forwarded with higher priority than the level-2 ones. Only when the level-1 queue is empty, packets in level-2 queue then can be sent. The path between source node 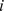 and destination 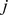 is denoted as



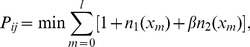
(3)where 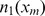 and 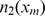 are the queue length of level-1 and level-2 on node 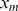 respectively, and 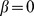 for level-1 and 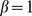 for level-2 when computing path. The first-in-first-out (FIFO) discipline is applied for each queue. Once one packet arrives at its destination, it is removed from the system. If there are more than one paths between two nodes with equal minimum cost, we randomly choose one.

The network traffic capacity can be measured by the maximal packet generation rate 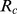, at which the phase transition switches from free flow state to congested flow state. It can be denoted by the order parameter [41]

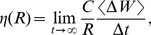
(4)where 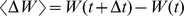, 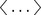 indicates the average time windows of width 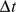, and 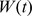 is the total packets’ number within the network at time 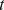. When 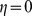, new generated packets and the removed packets are balanced, and no congestion occurs. With increasing packet generation rate 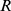, 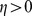, the inflow exceeds the outflow and some nodes in the network begin to be jammed.

## Simulation Results

For this two-level flow traffic model, we denote the fraction of level-1 packets as 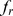, then portion 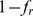 for level-2 packets. Level-1 packets are sent prior to level-2 packets. To do the comparisons, in this paper, we also apply the global shortest path routing strategy for the two-level flow traffic model. Firstly, we investigate the evolution of traffic capacity 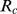 with different 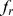 under the shortest path routing and dynamic source routing strategy. In our simulations, for each packet generation rate 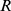, we do 10000 time steps to calculate the order parameter 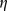 for two-level flow traffic model.

**Figure 1:**
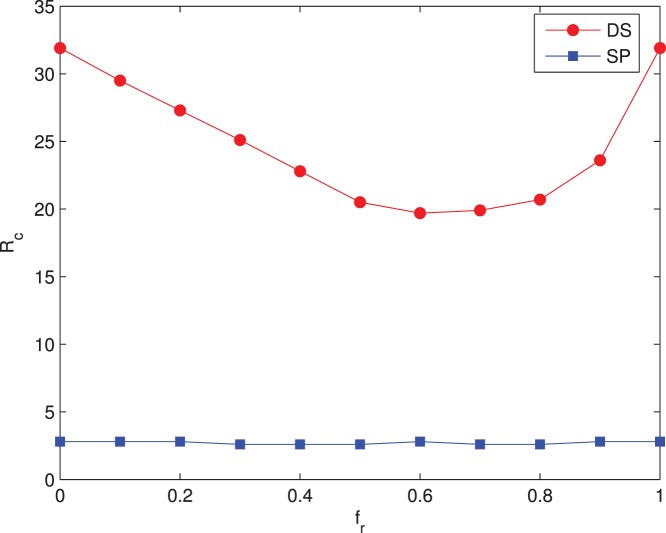
The critical packet generation rate 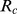 vs 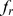 under different routing strategies. Network parameters: 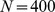, average degree 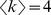. Curves with solid circles and solid squares represent traffic capacity 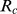 under DS and SP respectively.

As shown in Figure 1, when 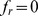 or 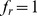, all packets are with the same level, and the traffic capacity 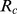 achieves the highest value 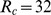 in our simulations. When 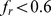, 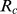 decreases with 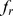; while when 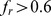, the traffic capacity increases with 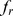. Under the global shortest path routing, 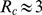 for all 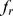. In many real complex networks, such as the Internet, in order to guarantee the quality of transmission of level-1 packets, 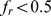 is more realistic and reasonable. Our results show that dynamic source routing can effectively enhance the traffic capacity compared with the shortest path routing.

As discussed in Ref. [9], the path one packet traverses through with the shortest length is not necessarily the quickest way, when considering the presence of possible traffic congestion and waiting time along the shortest paths. Queues of central nodes that one packet easily chooses to pass through to arrive at its destination are longer than other non-center nodes, and the waiting time for one packet on central nodes is much longer. Packets are delayed and thus the whole network get congested. Dynamic source routing strategy computes the path for each packet based on the current traffic load on each node. It can bypass the central nodes and shorten the waiting time of the packets.

**Figure 2:**
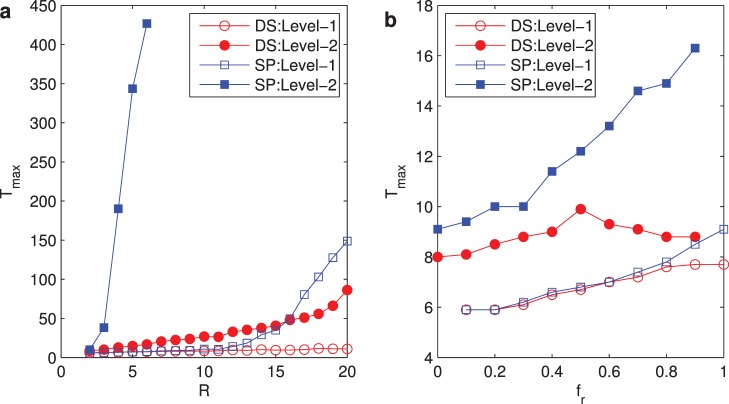
Variations of maximum packet traveling time 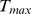. (a) 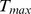 vs packet generation rate 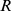 at 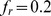; (b) 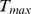 vs 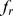 at 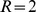. Network parameters: 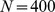, average degree 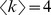. Curves with hollow circles and hollow squares represent 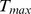 of level-1 under DS and SP respectively, and curves with solid circles and solid squares represent 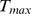 of level-2 under DS and SP respectively.

Traveling time of packets is also an important factor to character the network’s performance and here it is defined as the time steps that a packet takes to reach the destination from the source. To guarantee the efficiency of all level-1 time-sensitive packets, 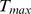 of all packets must not exceed a particular explicit value. In Figure 2a, we show the 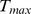 for two-level packets under the shortest path routing and dynamic source routing. With increasing packet generation rate 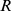, 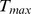 of level-1 packets under the dynamic source routing is the smallest, while the 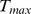 of level-1 packets under the shortest path routing(SP) is a little longer than that of DS when 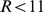. When 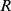 goes beyond 12, 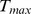 of level-1 packets under the SP is much larger than that of DS. 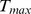 of level-2 time-insensitive packets under DS is larger than that of level-1, but dramatically shorter than that of level-2 under SP. When 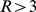, 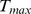 of level-2 packets under SP is relatively long, and part level-2 packets on central nodes have fewer opportunities to be forwarded in time. On the contrary, 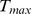 of level-2 packets under DS can be guaranteed within an upper limitation. In Figure 2b, we choose packet generation rate 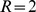 at which no network congestion happens under both DS and SP. Again, with increasing 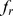, 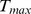 of level-1 packets under DS is shorter than that of level-1 packets under SP. 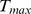 of level-2 packets under SP is the largest under all 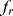. Combined Figure 2a and Figure 2b, dynamic source routing can better guarantee the transmission efficiency of level-1 time-sensitive packets. By bypassing the central nodes with heavy traffic load, traffic capacity of network is enhanced and time-sensitive flows are better supported under our proposed dynamic source routing strategy on two-level flow traffic model.

Under dynamic source routing strategy, the routing table is computed at each time step and it is time-consuming to compute the average path length of DS. For simplicity, here we compute the average path length by using average hops of all actual packets from their sources to destinations in our simulations.

**Figure 3:**
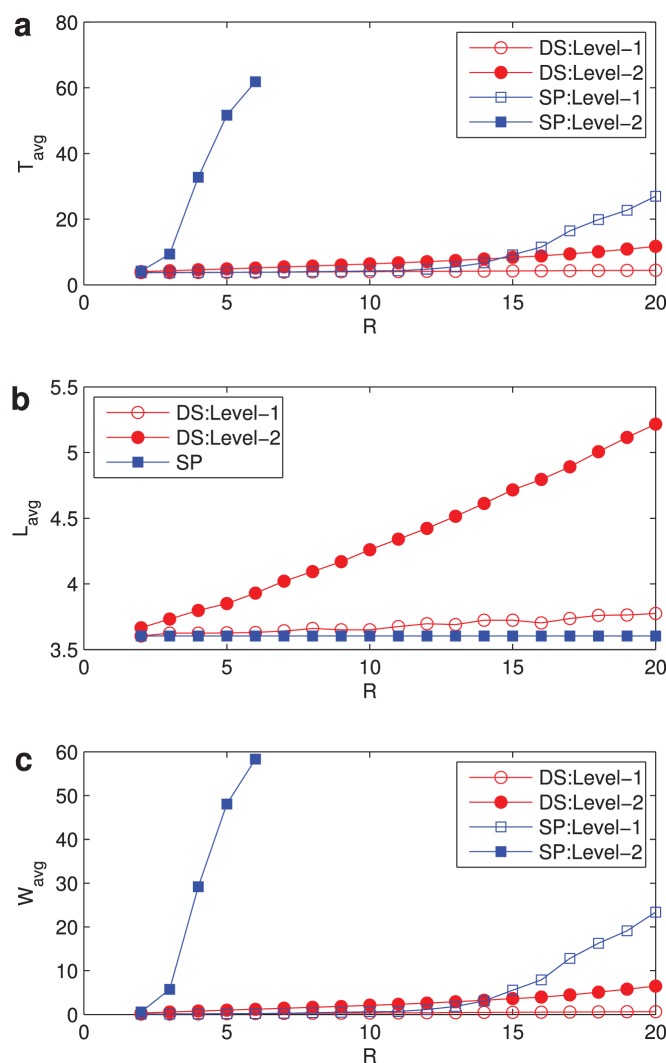
Variations of network metrics. (a) 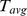 vs 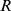; (b) 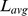 vs 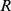; (c) 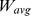 vs 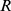 with 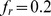. Network parameters: 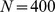, average degree 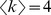. Curves with hollow circles and hollow squares represent the metric of level-1 under DS and SP respectively, and curves with solid circles and solid squares represent the metric of level-2 under DS and SP respectively.

**Figure 4:**
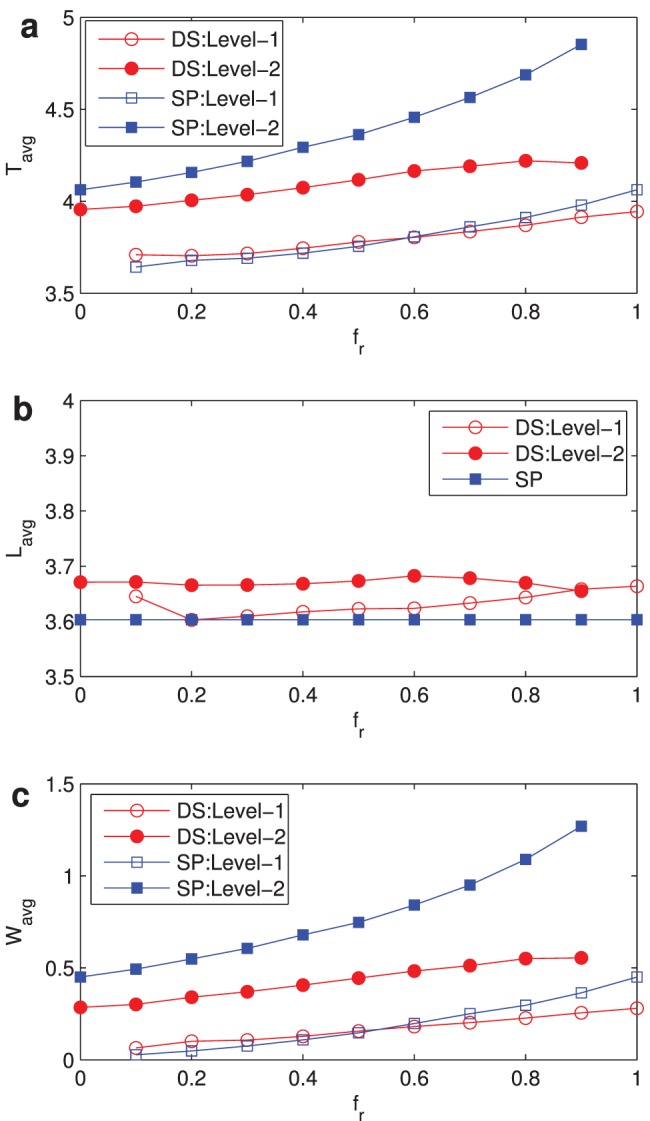
Variations of network metrics. (a) 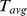 vs 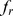; (b) 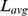 vs 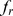; (c) 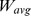 vs 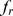. The packet generation rate 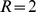. Network parameters: 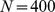, average degree 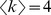. Curves with hollow circles and hollow squares represent the metric of level-1 under DS and SP respectively, and curves with solid circles and solid squares represent the metric of level-2 under DS and SP respectively.

In Figure 3, we investigate the evolutions of average traveling time 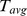, average path length 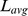 and average waiting time 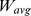 with 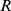 when 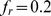. The relationship of these three metrics is: 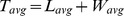. We observe that the average packet traveling time for level-1 packets under DS is the smallest for all 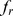, and the average packet traveling time for level-2 packets under DS is much smaller than that of level-2 packets under SP. In Figure 4, we evaluate the evolutions of these three metrics with 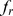, when 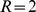 at which the network is in free flow state under both DS and SP routing strategies. Average packet traveling time for level-1 packets under both DS and SP is about the same. Definitely, average path length under the SP is the smallest. Meanwhile, the average path lengths under DS for two levels are different, and level-2’s is a bit longer than level-1’s. Average waiting time has similar evolutions to the average traveling time. Combined Figure 2, Figure 3 and Figure 4 with Figure 1, we can conclude that traffic capacity under DS for two-level flows is higher and time-sensitive packets can be sent to their destinations within limited time. DS on two-level flow traffic model can improve the network performance that the previous routing strategies can’t do, especially guaranteeing the delivery time of time sensitive flows such as telephone communications and video conferences.

**Figure 5:**
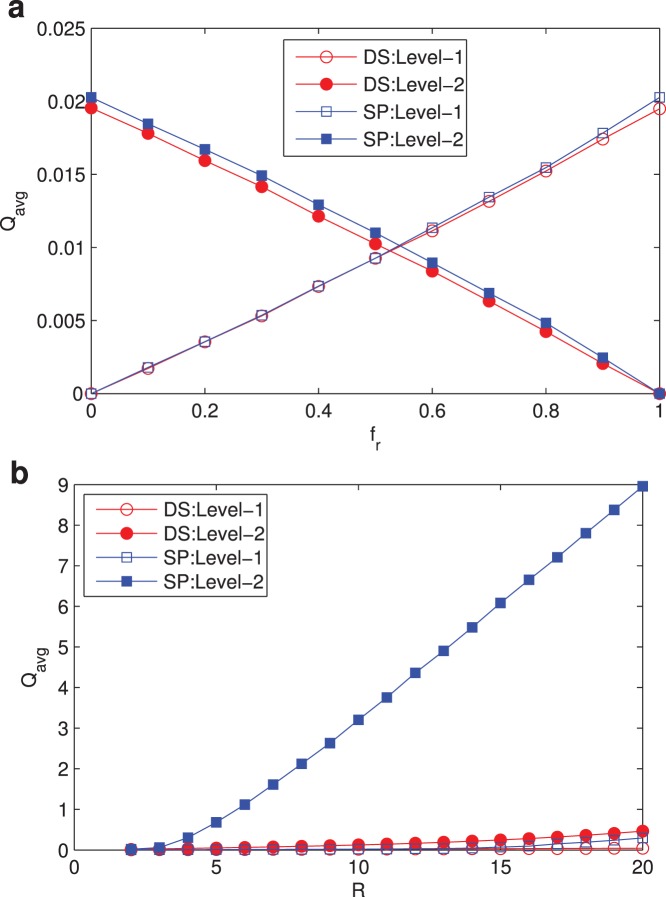
Variations of average queue length 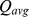 on all nodes. (a) 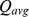 vs 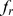 with 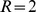; (b) 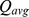 vs 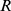 with 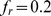. Network parameters: 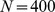, average degree 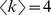. Curves with hollow circles and hollow squares represent 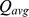 of level-1 under DS and SP respectively, and curves with solid circles and solid squares represent 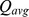 of level-2 under DS and SP respectively.

Traffic load of nodes in the network under different routing strategies is essential, for it reveals the utilization of all queue resources. Here, we evaluate traffic load by measuring the average queue length for two-level flows of all nodes under the two routing strategies. In Figure 5a, at 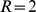, it is interesting that average traffic load of two levels under DS is smaller than that of SP under all 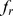. With increasing fraction 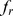 of level-1 packets, the portion of level-1 packets in the network grows, while the number of level-2 packets decreases. In Figure 5b, average traffic load on each node increases with the packet generation rate 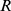. Level-2’s average traffic load increases sharply while the other three’s grow slowly. When 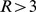, time-insensitive packets of level-2 under SP can’t be sent timely and tolerate long transfer time which might be acceptable. The more level-2 packets enter the network, the longer the level-2 queue grows under SP.

## Discussion

With consideration of real applications in which a part of them are time-sensitive and the others are time-insensitive, we proposed a two-level flow traffic model to satisfy different transmission needs. Packets of level-1 were delivered preferentially to level-2 to guarantee the quality of level-1 time-sensitive packet’s transition. To achieve high traffic capacity of network, we propose and investigate a new dynamic source routing. Path of one packet under dynamic source routing was included in the packet and the packet was delivered according to the path. Thus no routing table was used in the process of packet delivering, and it supported the global dynamic routing primely. With higher traffic capacity and supporting diverse traffic flows, our dynamic source routing has a good application perspective in real networks such as Ad-Hoc wireless networks or for next generation networks in future.

The dynamic source routing strategy on two-level flow traffic model expands the possibility of application of the global dynamic routing strategy. To our best knowledge, this work is the first one to differ the time-sensitive and time-insensitive flows to guarantee the real-time applications in complex systems.
